# Coronary stenting versus bypass surgery in elderly with multivessel disease: long-term mortality rate is still up for debate

**DOI:** 10.1007/s12471-020-01514-x

**Published:** 2020-11-10

**Authors:** T. L. Braber, R. S. Hermanides, J. P. Ottervanger

**Affiliations:** Department of Cardiology, Isala Hospital, Zwolle, The Netherlands

Dear editor,

Based on their long-term follow-up study in elderly patients with multivessel disease, Gimbel et al. reported a significantly higher 5‑year mortality after percutaneous coronary intervention (PCI) compared with coronary artery bypass grafting (CABG) in a recent issue of the *Netherlands Heart Journal* [1]. In addition, completeness of revascularisation appeared not to be an independent predictor of adverse outcomes in this patient population [1]. We wish to highlight a few factors that require clarification and may influence the differences observed between the study groups.

First and foremost, this is an observational study, with risk of confounding. Although the authors state in the statistical paragraph they adjusted for baseline variables with a *p*-value <0.1 in the univariate analysis, the results section only mentions adjustments for older age, higher creatinine levels and left main coronary artery disease (LMCAD), not for baseline differences such as gender, history of CABG, urgent status or complete revascularisation. Moreover, the authors failed to collect data on very important baseline predictors of mortality in elderly that also influence the decision for mode of revascularisation, such as cancer, neurological disorders (e.g. dementia) and frailty. Without adjusting for these variables, the conclusion that CABG is associated with lower mortality must be drawn with great caution.

Also, data on a history of late stent thrombosis with drug-eluting stents (DES) and the use of potent P2Y_12_ platelet receptor antagonists (ticagrelor, cangrelor) are lacking. In addition, in this observational study with the aim of assessing a treatment effect of CABG versus PCI but with imbalanced baseline characteristics, the authors used only Cox proportional hazard regression analysis instead of propensity score matching, which seems more appropriate as control for bias.

Although the 2007 BARI trial demonstrated suboptimal outcomes of PCI in patients with multivessel disease compared with CABG, especially in diabetic patients, more recent randomised trials comparing CABG and PCI with newer-generation DES and advanced medical therapy (as opposed to plain old balloon angioplasty in the BARI trial and first-generation DES in the FREEDOM trial) have shown fewer advantages of CABG over PCI, especially for LMCAD [2]. In addition, the controversial EXCEL trial recently demonstrated a nearly identical 5‑year hazard of death, myocardial infarction or stroke with PCI using a second-generation DES versus CABG in 1905 patients with LMCAD (mean age 66 years), 51% of whom had concomitant multivessel disease. However, although PCI showed superior 30-day outcomes, further analysis of mortality revealed a small but significant increase in death from any cause after 5 years for PCI versus CABG (odds ratio 1.38, 95% confidence interval 1.03–1.85), emphasising the importance of long-term follow-up.

Still, the development of new chronic total occlusion techniques and the introduction of enhanced guidewires, i.e. microcatheters combined with new devices, have raised the procedural success of chronic total occlusion to nearly 90% [3], and have reduced the ischaemic burden. Current intracoronary imaging techniques, such as intravascular ultrasound and optimal coherence tomography, can improve stenting results by providing detailed characterisation of the arterial wall and reveal stent findings complementary to those seen with angiography, thus leading to optimisation of PCI results in elderly patients.

In conclusion, we think that based on the data presented by Gimbel et al., it cannot be concluded that long-term mortality is higher in elderly patients with multivessel disease undergoing PCI compared with CABG.
